# Antigenic Relatedness between Mannans from *Coccidioides immitis* and *Coccidioides posadasii* Spherules and Mycelia

**DOI:** 10.3390/jof10020089

**Published:** 2024-01-23

**Authors:** Amanda R. Burnham-Marusich, Kathleen R. Zayac, John N. Galgiani, Lourdes Lewis, Thomas R. Kozel

**Affiliations:** 1 DxDiscovery, Inc., Reno, NV 89557, USA; 2 Department of Microbiology and Immunology, University of Nevada, Reno School of Medicine, Reno, NV 89557, USA; kzayac@unr.edu (K.R.Z.); tkozel@med.unr.edu (T.R.K.); 3 Valley Fever Center for Excellence, College of Medicine-Tucson, University of Arizona, Tucson, AZ 85721, USA; spherule@arizona.edu (J.N.G.); lulewis4@arizona.edu (L.L.); 4 Department of Medicine, College of Medicine-Tucson, University of Arizona, Tucson, AZ 85721, USA; 5 Department of Immunobiology, College of Medicine-Tucson, University of Arizona, Tucson, AZ 85721, USA; 6 BIO5 Institute, University of Arizona, Tucson, AZ 85721, USA

**Keywords:** mannan, immunoassay, *Coccidioides*, *Cryptococcus*, coccidioidomycosis, antigenic relatedness, diagnostics, antigen-detection immunoassay

## Abstract

Immunoassays for cell wall mannans that are excreted into serum and urine have been used as an aid in the diagnosis of many disseminated fungal infections, including coccidioidomycosis. Antigen-detection immunoassays are critically dependent on the detection of an analyte, such as mannan, by antibodies that are specific to the analyte. The goal of this study was to evaluate the extent of cross-reactivity of polyclonal antibodies raised against *Coccidioides* spp. Analysis of antigenic relatedness between mannans from *C. posadasii* and *C. immitis* spherules and mycelia showed complete relatedness when evaluated by the method of Archetti and Horsfall, which was originally used to study the antigenic relationships between Influenzae virus isolates. In a further effort to validate the suitability of the antigenic relatedness calculation methodology for polysaccharide antigens, we also applied the method of Archetti and Horsfall to published results that had previously identified the major capsular serotypes of *Cryptococcus* species. The results of this analysis showed that Archetti and Horsfall’s antigenic relatedness calculation correctly identified the major cryptococcal serotypes. Together, these results suggest that the method is applicable to polysaccharide antigens, and that immunoassays that detect *Coccidioides* mannans are likely to have good reactivity across *Coccidioides* species (inclusivity) due to the species’ high level of antigenic relatedness.

## 1. Introduction

Fungal cell-wall polysaccharides are well-established biomarkers for invasive fungal diseases caused by Ascomycete fungi. Specifically, mannan or galactomannan have been detected in blood or urine from patients with invasive candidiasis [1,2,3], aspergillosis [4,5], blastomycosis [6], fusariosis [7,8], histoplasmosis [9,10], and coccidioidomycosis [11]. In all the above diseases, the detection of shed mannan or galactomannan in the patient specimens was achieved by antigen-detection immunoassay.

The presence of galactomannan (Gm) in the urine of patients with coccidioidomycosis was first noted by Kuberski et al., who found that a *Histoplasma* antigen test obtained positive results with urine samples from patients with acute or chronic coccidioidomycosis [12]. Subsequent studies generated an immunoassay for the detection of *Coccidioides* Gm. In these studies, capture and detector antibodies were constructed using serum from rabbits that were immunized with a cocktail of purified *C. immitis* and *C. posadasii* Gm and formalin-killed mold [11]. The results showed the presence of *Coccidioides* Gm in the serum and urine of patients with coccidioidomycosis [11,13,14]. These studies also found that a small number of patients with *Coccidioides* infection received positive results with a *Histoplasma* antigen immunoassay. Conversely, a small number of patients with histoplasmosis received positive results with the *Coccidioides* antigen immunoassay.

The tissue form of *Coccidioides* spp. in patients is the spherule phase of the fungus, rather than the mycelial form that was used to develop the detection reagents for the original *Coccidioides* antigen immunoassay. Moreover, coccidioidomycosis is caused by two distinct species, *C. immitis* and *C. posadasii*. Successful diagnostics require high clinical sensitivity, for which high inclusivity across both *Coccidioides* species is a prerequisite. They also require high clinical specificity, meaning low cross-reactivity with other non-*Coccidioides* fungi. The goal of the present study was to assess the extent of cross-reactivity between mannans from the spherules and mycelia of *C. immitis* and *C. posadasii* using serum from immunized rabbits to further our understanding of how best to create diagnostics with the desired inclusivity. We validated the applicability of our methods for fungal carbohydrate antigens by analyzing raw titer data from the seminal studies that identified the major capsular serotypes of *Cryptococcus* species. Lastly, we also evaluated the reactivity of *Coccidioides* antisera with mannans from other selected fungi, including *Aspergillus fumigatus*, *Candida albicans,* and members of the Mucorales to determine what challenges a *Coccidioidies* antigen-detection immunoassay would have to surmount to obtain the desired specificity in the clinic.

## 2. Materials and Methods

### 2.1. Fungal Cultures

The following fungi were used: *Coccidioides immitis* (RS) and *Coccidioides posadasii* (Silveira) from Dr. John Galgiani at the University of Arizona; *Aspergillus fumigatus* Fresenius (CBS 101355 [AF 293]) from ATCC (Gaithersburg, MD, USA) #MYA-4609; *Candida albicans* (Robin) Berkhout (SC5314) from ATCC #MYA-2876; *Fusarium solani* (95-2478) and *Rhizopus oryzae* (99-892), both from Dr. Ashraf Ibrahim of the Lundquist Institute for Biomedical Innovation at Harbor-UCLA Medical Center; *Rhizopus oryzae* Went et Prinsen Geerligs (G) from ATCC #46599; *Mucor circinelloides* f. *circinelloides* Schipper (C81) from ATCC #8542; and *Mucor circinelloides* f. *circinelloides* Schipper (704) from ATCC #8097. All fungi except *C. immitis* and *C. posadasii* were cultured in RPMI-1640 (Caisson Labs, Smithfield, UT, USA), supplemented with 2% (*w/v*) glucose at 37 °C. *C. immitis* and *C. posadasii* saprobic-phase cells (mycelia) were grown by adding an arthroconidia spore suspension to a flask containing culture media (RPMI-1640 (Sigma-Aldrich, St. Louis, MO, USA) with L-glutamine and sodium bicarbonate); then, the flask was incubated at 37 °C, at 160 rpm for 96 h. For parasitic-phase cells (spherules), inoculation and growth were as described for mycelia, but the parameters for incubation were: 38 °C, 180 rpm, 20% CO_2_, and 96 h incubation.

### 2.2. Mannan Extraction, Purification and Analysis

Fungal mycelia and spherules were harvested by filtration (Whatman #1 filter paper) and centrifugation at 5525× *g*, respectively, and washed extensively with deionized, 0.22 μm filtered water. *Candida albicans* blastoconidia were harvested by centrifugation at 4000× *g* for 5 min and washed with water three times, centrifuging again after each wash. Mannans were extracted through the modification of the hot citrate method of Peat [15]. In brief, the washed fungal cells were collected, weighed, and resuspended in 10 volumes (*v/w*) of 0.1 M citrate buffer, pH 7.0. The fungal cell suspensions were autoclaved for 25 min. The autoclaved cells were harvested by 0.22 μm filtration or, in the case of *Candida* cells, by centrifugation, and the supernatant fluid was collected. The washed cells were resuspended in an additional 10 volumes of 0.1 M citrate buffer, pH 7.0 and autoclaved again for 25 min, after which the cells were again separated from the supernatant fluid, as above, and the supernatant fluid was collected. Mannan was purified from the pooled supernatant fluids via affinity chromatography on concanavalin A-Sepharose 4B, dialyzed against water, lyophilized, and weighed.

Mannan compositions were determined by the Complex Carbohydrate Research Center (University of Georgia, Athens, GA, USA). All samples were first subjected to acidic methanolysis. Glycosyl composition analysis was performed via a combined gas chromatography/mass spectrometry (GC/MS) analysis of the per-*O*-trimethylsilyl derivatives of the monosaccharide methyl glycosides. Protein concentration was determined using the Qubit™ protein assay kit (Invitrogen, Waltham, MA, USA).

### 2.3. Immunization of Rabbits

The immunization of rabbits for polyclonal antibody production was approved by the University of Nevada, Reno Institutional Animal Care and Use Committee (DxDiscovery IACUC protocol number 20-06-1026-1), and was performed in accordance with relevant guidelines and regulations. All immunizations and the collection of sera were carried out by ProSci, Inc. (Poway, CA, USA). To prepare antigens for the immunization of rabbits, *Coccidioides* mycelia and spherules were first inactivated by treatment overnight with 1% formalin at room temperature. Formalin-killed mycelia and spherules were washed with water and cultured to ensure inactivation. Fungal cells were then lyophilized, resuspended in endotoxin-free, sterile PBS, and lightly ground with a tissue grinder (Fisherbrand 02-542-09, Fisher Scientific, Waltham, MA, USA) until they could pass through a 22-gauge needle. The immunization protocol was adapted from previous hyperimmunization protocols that were used to generate antibodies to capsular polysaccharides of *Cryptococcus* species [16,17] and the Group A polysaccharide of *Streptococcus pyogenes* [18]. Rabbits were intravenously immunized with 0.5 mL of a 2 mg/mL solution of inactivated *Coccidioides* cells per dose. Doses were delivered every 2–3 days, for a total of 9 doses, which was followed by a rest period of 5 weeks and then a second round of 9 additional doses. Two rabbits were immunized with each antigen preparation. Two weeks following the final dose, animals were euthanized and exsanguinated, and serum was prepared.

### 2.4. ELISA for the Determination of Antibody Levels

Microtiter plates (655001, Greiner Bio-One, Monroe, NC, USA) were coated overnight with 100 μL of mannan at a concentration of 4 μg/mL in PBS. The plates were then washed with PBS containing 0.05% Tween-20 (PBS-T) and blocked for 90 min with blocking buffer (PBS containing 0.5% Tween-20 and 5% *w*/*v* powdered milk). The plates were washed with PBS-T and incubated for 90 min with serial dilutions of rabbit antisera in blocking buffer. The serum dilution series began at 1/4000 and extended through 16 two-fold dilutions. The plates were washed with PBS-T and incubated for 90 min with a 1/5000 dilution of peroxidase-labeled goat anti-rabbit IgG (H+L) (111-035-144, Jackson Immunoresearch, West Grove, PA, USA). Finally, the plates were washed with PBS-T, incubated for 30 min with substrate, and the reaction was stopped through the addition of 1M H_3_PO_4_. The absorbance of microtiter wells was read at 450 nm. Endpoint titers were calculated as the serum dilution that produced an OD = 0.5 in a log–log plot of serum dilution vs. OD.

### 2.5. Calculation of Antigenic Relatedness

Studies of antibody levels are reported as follows: (i) reciprocal antibody titer, (ii) titer ratios, and (iii) antigenic relatedness. Geometric mean titers from each immunization group were used to calculate titer ratios. Titer ratios were calculated as follows: [(geometric mean titer of serum against heterologous mannan)/(geometric mean titer of serum against homologous mannan)]. By definition, the homologous titer ratio is always 1. Antigenic relatedness (*r*) was calculated according to Archetti and Horsfall as *r* = (*r*_1_ × *r*_2_)^1/2^, where *r*_1_ is the titer ratio of the heterologous titer obtained with fungus 2 to the homologous titer obtained with fungus 1 and *r*_2_ is the titer ratio of the heterologous titer obtained with fungus 1 to the homologous titer obtained with fungus 2.

## 3. Results

An initial experiment compared the glycosyl composition and protein content of mannans isolated from *C. posadasii* and *C. immitis* mycelia and spherules. The analysis found that all mannans were composed of 3-*O*-methyl mannose, galactose, mannose, and glucose (Table 1). The 3-*O*-methylmannose content was approximately three times higher in spherules than in mycelia. Previous studies found that 3-*O*-methyl mannose was a constituent of extracts from the mycelia of *C. immitis* [19,20].

The immunization of rabbits with whole mycelia or whole spherules from either *C. posadasii* or *C. immitis* led to very high titers of serum antibodies to purified mannans. Every rabbit produced reciprocal titers that exceeded 10^6^. The serum antibody titers for each rabbit in each immunization group, e.g., *C. posadasii* (*Cp*) mycelium, *C. posadasii* spherules, *C. immitis* (*Ci*) mycelium, and *C. immitis* spherules, are shown in Table 2. The results showed extensive cross-reactivity among the various *Coccidioides* mannans.

The extent of the reciprocal cross-reactivity between the various mannans was determined by the methods of Archetti and Horsfall [21]. In this analysis, the titer ratios for various antisera were calculated as the titer with each heterologous mannan divided by the titer with the homologous mannan (Table 3). A single value that represented the antigenic relationship between mannans from *C. immitis* or *C. posadasii* mycelia or spherules was then calculated (Table 4). The results of the antigenic relatedness calculation showed near-identical antigenic relatedness with all antisera and mannans, with relatedness values that ranged from 0.9 to 1.5.

Given the apparent lack of antigenic heterogeneity found with mannans from *C. posadasii* and *C. immitis* mycelia and spherules when titers were evaluated using the method of Archetti and Horsfall, we revisited the original reports that identified cryptococcal serotypes A, B, C, and D from 1950 and 1968 [16,17]. The goal was to determine whether the antigenic relatedness calculation of Archetti and Horsfall correctly identified significant antigenic differences among the polysaccharide cryptococcal serotypes that established the four major cryptococcal serotypes. The original studies provide cross-reactivity titers from the sera of rabbits immunized with whole cells of *Cryptococcus neoformans*. The results from a retrospective analysis of the published cross-reactivity titers showed that the antigenic relatedness calculation using the method of Archetti and Horsfall correctly identified all the distinct cryptococcal serotypes where the classical agglutination of cross-absorbed sera was used for the determination of serotypes (Table 5 and Table 6).

A final experiment examined the reactivity of the antisera raised against *C. posadasii* and *C. immitis* mycelia and spherules with mannans of selected fungi. The results (Table 7) showed the extensive reactivity of antisera from rabbits immunized with *C. posadasii* or *C. immitis* mycelia with mannans of *Aspergillus*, *Fusarium*, *R. oryzae* 46599, and *M. circinelloides* 8542. A much lower reactivity was found for the antisera from mycelia-immunized rabbits with mannans of *C. albicans*, *R. oryzae* 99-892, and *M. circinelloides* 8097. The patterns of cross-reactivity induced by mycelia were similar, regardless of whether rabbits were immunized with *C. posadasii* or *C. immitis*. In contrast to the considerable cross-reactivity of sera from animals immunized with mycelia, there was very limited reactivity in the antisera from spherule-immunized rabbits with the various non-*Coccidioides* mannans.

## 4. Discussion

Coccidioidomycosis is a common cause of community-acquired pneumonia in endemic regions such as southern Arizona and the San Joaquin Valley in California [22]. Approximately 20,000 cases are reported in the U.S. each year, but preliminary studies from the CDC suggest that the actual burden of symptomatic cases may be 6-14 times higher [23]. However, climate change will likely expand the endemic region to include large portions of the western United States [24]. In addition, the number of cases will also increase due to population growth in endemic regions and advances in healthcare that will expand the at-risk population [25].

As the number of cases increases, there will be a need for more effective diagnosis and treatment. Currently, serologic tests are the most commonly used methods for the diagnosis of coccidioidomycosis [26,27]. A limitation to the use of serology is the lag between symptom onset and antibody production [25,27]. In addition, the sensitivity of serology may be lower in immunosuppressed patients [25]. As a consequence, there is an increased interest in antigen detection, which may detect infection at early stages of the disease [11,12,14]. Antigen detection is also useful in the diagnosis of infection in immunocompromised patients. The fungal antigen detected in antigen-detection immunoassay is the cell-wall mannan. A limitation to antigen detection is its low sensitivity in non-immunocompromised patients and cross-reactivity with other dimorphic fungi.

There is an increasing interest in simplifying immunoassays for the diagnosis of coccidioidomycosis. In particular, serologic tests using defined reagents for quantitative enzyme-linked immunoassays and use of the lateral flow immunoassay platform will make serological testing more accessible [28,29,30]. Similarly, the use of an immunoassay for the detection of cell-wall mannan in serum and urine using lateral flow can move laboratory testing closer to the point of patient care [23]. To this end, a major goal in our laboratory is the development of antigen immunoassays in lateral flow format, with increased analytical sensitivity and improved specificity.

The goal of this study was to determine the extent of antigenic relatedness between mannans from mycelia and spherules from representative strains of *C. immitis* and *C. posadasii*. The answer is important because immunoassays aiming to diagnose coccidioidomycosis using antigen-detection immunoassays will require: (i) reactivity with mannans from the tissue phase of the fungus, and (ii) an absence of bias in the immunological recognition of mannans from the two species. The results showed that, regardless of the *Coccidioides* immunogen used, the resulting antisera had complete reactivity across mannans from the two lifeforms and the two major genera (Table 4). This immunologic similarity occurred despite the considerable compositional differences between the mannans (Table 1).

The complete reactivity that is obtained across the *Coccidioides* mannans likely reflects very strong antigenic relationships, as shown by the use of antisera from immunized rabbits. An alternative explanation is that the antigenic relatedness calculation of Archetti and Horsfall fails to recognize antigenic differences in polysaccharides from pathogenic fungi. Antigenic relatedness was originally used to identify differences between Influenzae viruses and was subsequently used to study the relationships between a variety of viruses.

In an effort to address the ability of the antigenic relatedness calculation to identify antigenic differences among fungal polysaccharides, we reevaluated the original data that first identified the presence of serotypes A, B, and C of *Cryptococcus* species [16], as well a subsequent report of serotype D [17]. The results (Table 5 and Table 6) showed that the antigenic differences found by the calculation of antigenic relatedness correctly identified all four distinct serotypes found by the use of whole-cell agglutination with cross-adsorbed serum. The congruence of results using these two methods demonstrated that antigenic relatedness can be used with ELISA titers where polysaccharides are used as antigens.

Mannans from spherules and mycelia from *C. immitis* and *C. posadasii* are composed of similar carbohydrates, but the quantitative ratios are quite different. All four mannans had mannose and glucose as lesser components (<10%). Galactose (49–73%) and 3-*O*-methylmannose (10–44%) were major components. Previous studies found very similar compositions in crude extracts from *C. immitis* arthrospores, mycelium, and spherule cell walls [31]. Our results found the 3-*O*-methylmannose composition to be three times higher in mannan from *C. immitis* and *C. posadasii* spherules than mannan from mycelia, reflecting a likely fundamental difference in the cell-wall compositions of the two life forms. 3-*O*-methylmannose is an unusual carbohydrate that has been reported as a constituent in polysaccharides from *Mycobacteria* [32,33] and *Coccidioides* [19,20,31,34].

Our study evaluated antigenic relatedness as one step in the validation of mannan as a target for an antigen detection immunoassay. Serology has been extensively used as an effective tool in the diagnosis of coccidioidomycosis. We do not know the extent to which the mannans used in the present study would be recognized via the sera from patients with coccidioidomycosis. Nevertheless, our studies demonstrate that the mycelia and spherules of the two species have mannan antigens with close antigenic similarity. This similarity may prove important in further studies of antigen or antibody detection in clinical specimens. 

Antigenic relatedness was introduced by Archetti and Horsfall, who used a comparison of reciprocal antibody titers to evaluate the antigenic relatedness of various strains of Influenza A virus [21]. Since that time, antigenic relatedness has been used to analyze and predict vaccine efficacy, and to assess antigenic drift for Influenza virus and other viruses [35,36,37,38,39]. In this type of analysis, the serum titers raised against individual viruses are compared with the homologous immunizing antigens, as well as heterologous antigens. Antigenic relatedness values of 0.5 or less may identify significant differences, e.g., reference [38]. The application of this analysis to antigenic relatedness among mannans of *Coccidioides* found relatedness values that ranged from 0.9 to 1.5, suggesting a high degree of antigenic similarity between mannans of *C. posadasii* and *C. immitis* spherules and mycelia (Table 4), despite the considerable variability in the 3-*O*-methylmannose content of mannans from mycelia vs. spherules.

Previous studies of immunoassays for *Coccidioides* mannan in body fluids utilized antigen-detection ELISAs that were constructed using sera from rabbits immunized with *C. immitis* and *C. posadasii* galactomannan and mycelia. The extent of cross-reactivity with mannans of other fungi was not reported. However, the results in Table 7 suggest that the specificity of immunoassays constructed from polyclonal rabbit antisera would be optimized by the use of antisera from rabbits immunized with spherules alone. An alternative approach would be construction of antigen immunoassays using monoclonal antibodies of the desired specificity. We are currently working on this approach.

## Figures and Tables

**Table 1:** Composition of purified mannans from different species and growth forms.

*Coccidioides* Species and Growth Form	Glycosyl Composition (%) ^a^					Protein ^a^ (%)
	Arabinose	Galactose	Mannose	Glucose	3-*O*-Me-Mannose	
*C. posadasii* mycelia	0.3	63	3.3	15	17	8.6
*C. posadasii* spherules	-	49	1.9	5.2	44	11
*C. immitis* mycelia	1.8	73	8.1	6.6	9.9	4.4
*C. immitis* spherules	1.2	65	2.2	3.7	28	7.5

^a^ Glycosyl composition and protein, reported as the percentage of carbohydrate or protein present in dry samples.

**Table 2:** Cross-reactivity between *Coccidioides* mannans by ELISA.

Antiserum ^a^	Rabbit Number	Mannan ^b^			
		*Cp* Mycelia	*Cp* Spherules	*Ci* Mycelia	*Ci* Spherules
*C. posadasii* mycelia	Ra1	7850	5220	18,200	6460
	Ra2	5590	13,900	14,100	16,800
*C. posadasii* spherules	Ra3	3100	2600	3430	3030
	Ra4	7560	5970	9110	6770
*C. immitis* mycelia	Ra5	13,200	9920	20,200	11,100
	Ra6	10,600	8810	13,400	9690
*C. immitis* spherules	Ra 7	13,400	11,600	23,100	12,400
	Ra 8	12,000	6770	9790	6560

Results are expressed as endpoint titers in ELISA × 10^−3^. Results are titers from sera of individual rabbits (*n* = 2 rabbits per immunization group). ^a^ Antisera were obtained from rabbits hyperimmunized with the indicated fungal cells. ^b^ ELISA plates were coated with the indicated, purified fungal mannans.

**Table 3:** Titer ratios showing cross-reactivity between *Coccidioides* mannans by ELISA.

Antiserum	Mannan			
	*Cp* Mycelia	*Cp* Spherules	*Ci* Mycelia	*Ci* Spherules
*C. posadasii* mycelia	1.0	1.3	2.4	1.6
*C. posadasii* spherules	1.2	1.0	1.4	1.1
*C. immitis* mycelia	0.7	0.6	1.0	0.6
*C. immitis* spherules	1.4	1.0	1.7	1.0

Titer ratios are calculated as (titer of serum against heterologous mannan)/(titer of serum against homologous mannan) [21]. Calculations based on geometric means from ELISA titers shown in Table 2.

**Table 4:** Antigenic relatedness between *Coccidioides* mannans by ELISA.

Antiserum	Mannan			
	*Cp* Mycelia	*Cp* Spherules	*Ci* Mycelia	*Ci* Spherules
*C. posadasii* mycelia	1.0			
*C. posadasii* spherules	1.2	1.0		
*C. immitis* mycelia	1.3	0.9	1.0	
*C. immitis* spherules	1.5	1.0	1.0	1.0

Antigenic relatedness calculated from data in Table 2 using the method of Archetti and Horsfall.

**Table 5:** Antigenic relationship between cryptococcal serotypes A, B, and C, calculated from Evans [16].

**Cross-reactivity between cryptococcal isolates by tube agglutination titers**			
**Antiserum**	**Cells used for agglutination**		
	**Type A**	**Type B**	**Type C**
Type A (RE)	320	40	10
Type B (1523)	320	320	10
Type C (LE)	40	40	320
**Titer ratios showing cross-reactivity between cryptococcal serotypes**			
**Antiserum**	**Cells used for agglutination titers**		
	**Type A**	**Type B**	**Type C**
Type A	1.0	0.125	0.03
Type B	1.0	1.0	0.03
Type C	0.125	0.125	1.0
**Antigenic relationship between cryptococcal serotypes**			
	**Type A**	**Type B**	**Type C**
Type A	1.0		
Type B	0.35	1.0	
Type C	0.06	0.06	1.0

Agglutination titer data, as adapted from Evans [16]. Titer ratios were calculated from agglutination titers, as described in Table 3. Antigenic relationships were calculated, as illustrated in Table 4.

**Table 6:** Antigenic relationship between cryptococcal serotypes A, B, C, and D, calculated from Wilson, Bennett, and Bailey [17].

**Cross-reactivity between cryptococcal isolates obtained by slide agglutination titers**				
**Antiserum**	**Cells used for agglutination titers**			
	**Type A**	**Type B**	**Type C**	**Type D**
Type A (68)	1024	8	4	128
Type B (112)	1024	2048	256	512
Type C (18)	128	64	128	16
Type D (52)	256	256	32	256
**Titer ratios showing cross-reactivity between cryptococcal serotypes**				
**Antiserum**	**Cells used for agglutination titers**			
	**Type A**	**Type B**	**Type C**	**Type D**
Type A	1.0	0.008	0.004	0.125
Type B	0.5	1.0	0.125	0.25
Type C	1.0	0.5	1.0	0.125
Type D	1.0	1.0	0.125	1.0
**Antigenic relationship between cryptococcal isolates**				
	**Type A**	**Type B**	**Type C**	**Type D**
Type A	1.0			
Type B	0.06	1.0		
Type C	0.06	0.25	1.0	
Type D	0.35	0.5	0.125	1.0

Agglutination titer data were adapted from Wilson, Bennett, and Bailey [17]. Titer ratios were calculated from agglutination titers, as described in Table 3. Antigenic relationships were calculated as illustrated in Table 4.

**Table 7:** Reactivity of antisera raised against *C. posadasii* and *C. immitis* mycelia and spherules with mannans of other fungi.

Antiserum ^b^	Mannan Reactivity by ELISA (Reciprocal Titers × 10^−3^) ^a^							
	Homologous *Cp* or *Ci* Mannan ^c^	*Af* 4609	*Fs* 95-2478	*Ca* 2876	*Ro* 99-892	*Ro* 46599	*Mc* 8542	*Mc* 8097
*C. posadasii* mycelia	6630	432	1750	80	51	827	1150	52
*C. posadasii* spherules	3940	36	135	20	10	77	121	8
*C. immitis* mycelia	16,400	396	1640	47	30	425	742	115
*C. immitis* spherules	9020	133	503	10	23	156	177	15

^a^ *Cp*, *C. posadasii*; *Ci*, *C. immitis*; *Af*, *Aspergillus fumigatus*; *Fs*, *Fusarium solani*; *Ca*, *Candida albicans*; *Ro*, *Rhizopus oryzae*; *Mc*, *Mucor circinelloides*. Strain number is indicated for each fungus. ^b^ Antisera were obtained from rabbits hyperimmunized with the indicated *Coccidioides* cells. Results are the geometric mean of the sera of two rabbits. ^c^ Homologous mannan—ELISA was carried out using mannan from the immunizing antigen.

## Data Availability

Data are contained within the article.
